# Desmopressin in combination with anticholinergic agents in the treatment of nocturnal enuresis: a systematic review and meta-analysis

**DOI:** 10.3389/fped.2023.1242777

**Published:** 2023-10-19

**Authors:** Tong Cai, Yi Yao, Weigui Sun, Peipei Lei

**Affiliations:** ^1^ Department of Urology, The Affiliated Hospital of Yangzhou University, Yangzhou University, Yangzhou, China; ^2^ Department of Paediatrics, Yantai Yuhuangding Hospital, Yantai, China; ^3^ Department of Endocrinology, Yantai Yuhuangding Hospital, Yantai, China

**Keywords:** nocturnal enuresis (NE), desmopressin, anticholinergic agent, randomized controlled trials, meta-analysis

## Abstract

**Background:**

The desmopressin combined with anticholinergic agents for the treatment of nocturnal enuresis (NE) remains controversial. This meta-analysis assesses the efficacy and safety of desmopressin compared with desmopressin plus anticholinergic agents for the treatment of NE.

**Methods:**

We searched MEDLINE, Embase, and Cochrane Controlled Trials Register databases for RCTs published for the treatment of NE. Systematic review was carried out using the Preferred Reporting Items for Systematic Reviews and Meta-analyses. This meta-analysis used RevMan v.5.1.0 to analyze data.

**Results:**

Eight studies involving 600 patients (293 in the combination group and 307 in the desmopressin group) contained meaningful data. The results were as follows: after one month of treatment, compared with the desmopressin monotherapy group, the combination group was significantly better in treating NE in FR (full responders, *P* = 0.003), FR + PR (partial responders) (*P* < 0.0001), and the mean number of wet nights (*P* = 0.004); also, the combination group had a better effect in FR (*P* < 0.00001), FR + PR (*P* = 0.02) and the mean number of wet nights (*P* = 0.04) after 3 months' treatment. For side effects, combination therapy does not cause more adverse events in treating NE (*P* = 0.42).

**Conclusions:**

This study elucidates that desmopressin combined with the anticholinergic agent was demonstrated to be more effective in treating NE than desmopressin monotherapy, and the anticholinergic agent does not increase the risk of adverse events (AEs).

## Introduction

The International Children's Continence Society (ICCS) defines nocturnal enuresis (NE) as intermittent incontinence of urine or bed wetting during sleep in children aged >5 years (1, 2). According to the characteristics of NE, it can be categorized into primary and secondary enuresis. Primary enuresis is diagnosed when the symptoms of enuresis have persisted since infancy, while secondary enuresis is defined as a return to bedwetting after the patient has been consistently dry for at least 6 months at night. Moreover, based on the presence of daytime lower urinary tract symptoms (LUTS), enuresis can be categorized into monosymptomatic (MNE) or non-monosymptomatic (NMNE) (3). NE affects approximately 15%–20% of 5-year-old children, 5% of 10-year-olds, 1%–2% of individuals aged 15 years, and up to 2% of young adults (4, 5). It has brought a significant influence and considerable distress to the patients and their family. It would seriously affect the everyday life. The etiology and pathogenesis of NE are still unknown. However, in view of the present research, the primary etiology includes the following aspects: nocturnal low bladder capacity, nocturnal polyuria, and arousal disorder (6). At present, NE is not considered a self-healing disease, and it necessitates appropriate diagnostic and therapeutic work-up by humans. In principle, NE treatment can be divided into drug and non-drug treatment. The medication for NE includes desmopressin, anticholinergic drugs (tolterodine and solifenacin), and others (imipramine). Non-drug therapy includes bedwetting alarms and limitations of fluid intake (7).

Desmopressin, a naturally produced vasopressin analog, can increase the water permeability of collecting ducts and reduce urination frequency and nocturia (excessive urination at night) (8). At present, desmopressin is used as a first-line therapy for NE. It has been approved to cure NE (9). It has an immediate effect on NE and can be administered intranasally or orally. Compared with intranasal administration, oral administration has fewer side effects and is more convenient. The recommended dosage of desmopressin is 0.2 mg daily (taken orally). Faraj et al. (10) reported that desmopressin resulted in 85% dry nights after 3 months of treating MNE. However, research shows that approximately 20% of patients experience poor effects after drug therapy for 3 months, which is known as desmopressin-resistant NE (11). Moreover, functional bladder capacity decreases because of detrusor overactivity, the major cause of NE (12). However, in the clinical course of treatment, using a single drug (desmopressin monotherapy) cannot produce satisfactory results for patients with NE. Therefore, combination drug therapy was gradually used in clinical treatment and was proven efficient. Anticholinergic agents, such as tolterodine and solifenacin, may improve the function of the bladder and allow it to store more urine (13). Some studies show that the combined use of anticholinergic agents and desmopressin plays an important role in treating desmopressin-resistant NE, reducing the mean number of wet nights in patients with NE (14, 15). However, desmopressin combined with anticholinergic agents for treating NE remains controversial.

We performed a systematic review and meta-analysis of randomized controlled trials (RCTs) to assess the efficacy and safety of desmopressin compared with anticholinergic agents for treating NE.

## Materials and methods

### Search strategy

We searched the MEDLINE, Embase, and Cochrane Controlled Trials Register databases for RCTs published before January 2023 using the following search criteria: NE, desmopressin, anticholinergic agent, and RCT. We confined our search to published studies in English only and obtained certain essential information directly from the authors. We also screened the relevant references of the included studies.

### Inclusion criteria

**Figure 1:**
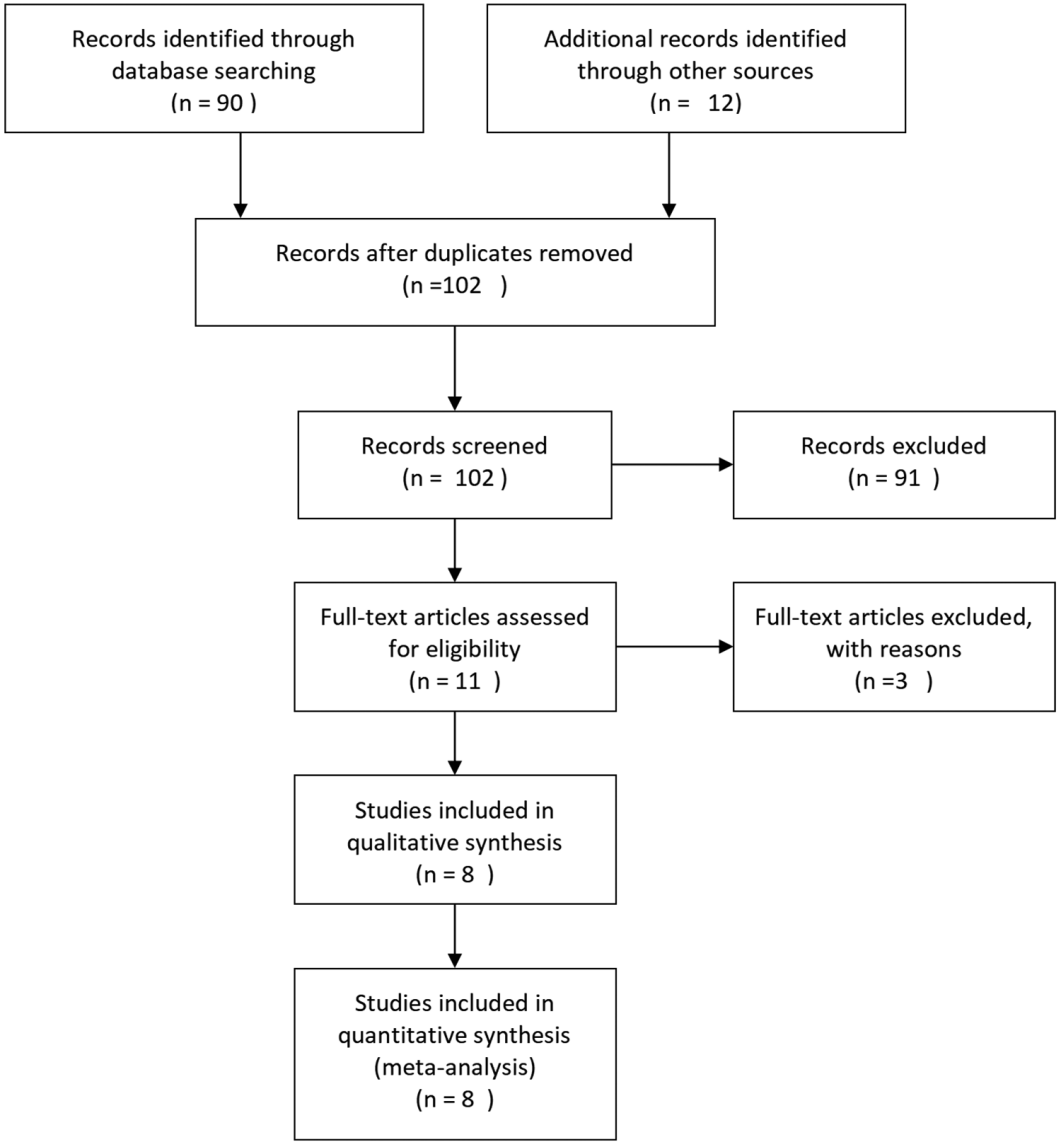
Flowchart of the study selection process.

All of the included RCTs meet the following criteria: (1) involved the use of desmopressin and anticholinergic agents for the treatment of NE, (2) had full useful RCT texts, and (3) provided accurate data for analysis, including the total number of subjects and the values of each indicator. The exclusion criteria include (1) data incompleteness in articles and (2) article types of abstract, review, case–control, comment, cohort studies, and others. We included the most recently published study only if it described identical experiments. However, each study would be included if different indicators were evaluated. As shown in Figure 1, we used a flowchart to show the selection process of the study.

### Quality assessment

**Figure 2:**
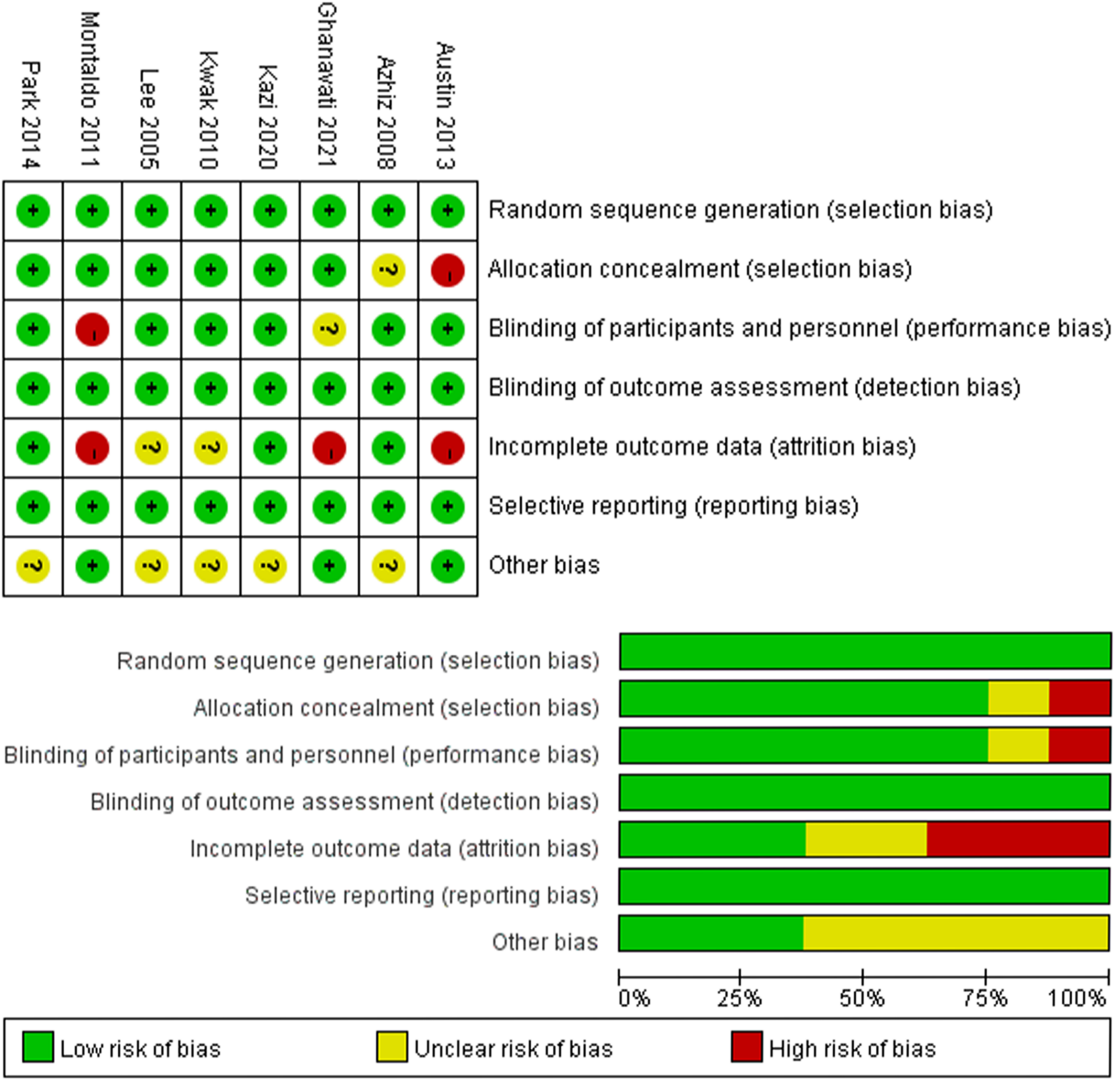
Risk of bias summary and graph.

The Cochrane risk of bias tool was used to determine the quality of the retrieved RCTs. The quality items were selective outcome reporting, blinding, allocation concealment, incomplete outcome data, random sequence generation, and other sources of bias. According to the discussions among the authors, a graph summarizing the risk of bias was generated, as shown in Figure 2. Meanwhile, according to the guidelines published in the Cochrane Handbook for Systematic Reviews of Interventions v.5.3.0, the studies were classified qualitatively (16). All authors participated in the quality assessment of all RCTs and agreed with the results. Moreover, any differences between each RCT were resolved through discussion among authors. All authors participated in the RCTs’ quality assessment and agreed on the final results.

### Data extraction

We recorded the following information from the studies: (1) general data of RCTs, (2) name of the first author, (3) design of the study and size of the sample, (4) published time, and (5) changes of efficacious data at 1 and 3 months for the parameters full responders (FR: ≥90% reduction in the number of wet nights), FR plus partial responders (PR: 50%–89% reduction in the number of wet nights), mean number of wet nights, and side effects. Finally, another author checked the data extracted from the text. Meanwhile, our team crosschecked the references and data for each included study to ensure no overlapping data and maintain the integrity of the meta-analysis.

### Statistical analysis and meta-analysis

This meta-analysis used RevMan v.5.1.0 (Cochrane Collaboration, Oxford, UK) to analyze the data (17). The odds ratio (OR) with 95% confidence interval (CI) was applied to analyze the dichotomous data, and the mean difference (MD) with 95% CI was utilized to analyze the continuous data among the groups. The chi-squared test based on *Q* statistic was performed to check the heterogeneity among the studies, and the result was recognized as significant at *P* < 0.05. The fixed-effects model was used and considered homogeneous if the result has a *P*-value of >0.05. We utilized the *I*^2^ statistic to analyze inconsistent results, which can reflect the proportion of heterogeneity across trials. When *I*^2^ < 50%, indicating that there was no significant heterogeneity, the fixed-effects model (Mantel–Haenszel method) would be used. We performed the random-effects model (DerSimonian and Laird method) when the heterogeneity of the data could not be explained (*P* < 0.05, *I*^2^ > 50%). In this meta-analysis, it is not necessary to have ethical approval and patient consent because all data were acquired from articles that have already been published.

## Results

### Characteristics of the individual studies

We identified 102 studies in all databases. According to the inclusion and exclusion criteria described above, our research removed 91 studies after reviewing the titles and abstracts of the articles. Three studies were excluded for lack of valuable data. A total of 26 studies were ruled out for lack of useful data. Finally, eight RCTs (18–25) involving 600 patients were included in our analysis. Figure 1 presents a detailed flowchart showing the selection process. Table 1 shows the baseline characteristics of the studies.

**Table 1:** Study and patient characteristics.

Study	Country	Therapy in experimental group	Therapy in control group	Sample size		Method	Time of therapy (weeks)	Dosage (mg/mg)	Type of enuresis	Main inclusion criteria
				Experimental	Control					
Austin 2008	USA	D + LAT	D + Pl	18	16	Oral	4	0.6 mg daily (D)/4 mg daily (LAT)	PNE	Aged 6–17 years, no LUT symptoms or bowel elimination problems, no episodes of daytime incontinence, no increased or decreased voiding frequency, no encopresis or constipation
Azhiz 2008	Iran	D + O	D	10	16	Oral	≥4	0.1 mg daily (D)/5 mg daily (O)	PNE	Aged 6–12 years; no urological, cardiovascular, and neurological diseases; no urinary tract infection and hypercalciuria; did not use any drug or conditioning therapy
Ghanavati 2021	Iran	D + T/S	D	40	22	Nasal administration/oral	≥4	1 puff once daily (D)/2 mg (T)/5 mg daily (S)	PMNE	Aged 5–15 years, primary nocturnal enuresis, wet at least four times over 4 weeks, no neurological or urological cause for enuresis
Kazi 2020	Pakistan	D + O	D	42	42	Oral	≥4	0.2 mg daily (D)/5 mg daily (O)	NE	Aged 7–13 years; no congenital anomalies, seizures, abnormalities of the central nervous system, diabetes insipidus, urinary tract infections, or any other co-morbidities
Kwak 2010	Korea	D + O	D	25	64	Oral	4	0.2–0.4 mg daily (D)/5–15 mg daily (O)	PRNE	No neuropathic bladder, no spinal dysraphism, no anatomical abnormalities and others
Lee 2005	Korea	D + O	D	48	49	Oral	≥4	0.1 mg or 0.2 mg daily (D)/5 mg daily (O)	PNE	Aged 6–15 years, ≥3 wet nights weekly, did not use any drug that affects nocturnal enuresis; no urinary tract infections or other organic urological diseases
Montaldo 2011	Italy	D + O	D + Pl	61	59	Oral	4	0.24 mg daily (D)/5 mg daily (O)	PMNE	Aged 6–13 years; no episodes of daytime incontinence, no increased or decreased voiding frequency, no encopresis or constipation
Park 2014	Korea	D + Pr	D	49	49	Oral	≥4	0.2 mg daily (D)/10 mg daily (Pr)	PMNE	Aged 5–16 years; no LUT symptoms or bowel elimination problems; no medical history of treatment for LUT symptoms or bowel elimination problems (encopresis or constipation); no medical history of treatment for OAB and others

D, desmopressin; O, oxybutynin; Pr, propiverine; Pl, placebo; LAT, long-acting tolterodine; T, tolterodine; S, solifenacin; OAB, overactive bladder; PNE, primary nocturnal enuresis; PMNE, primary monosymptomatic nocturnal enuresis; PRNE, pharmacotherapy-resistant NE.

### Quality of the individual studies

**Figure 3:**
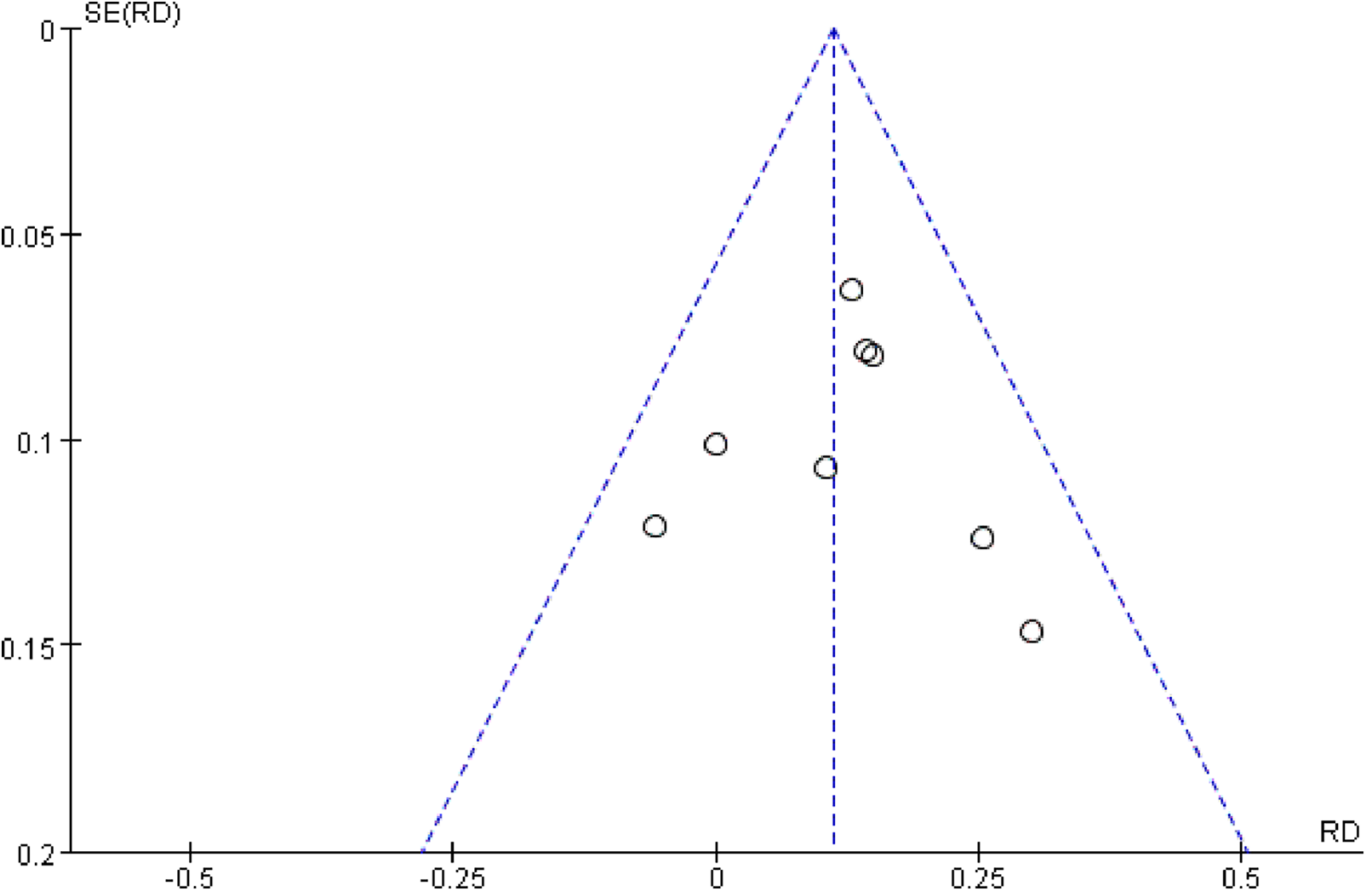
Funnel plot of the studies included in our meta-analysis. SE, standard error.

All eight studies were RCTs and double-blind. Figure 2 presents a graphical summary of the risk bias. Meanwhile, we also found that their randomization process had been elaborated in all the papers. All the included RCTs calculated the efficiency and determined the best sample size. The funnel plot displayed the conclusion of a qualitative estimation of the publication bias of each RCT (Figure 3). Table 2 shows the specific inclusion and exclusion criteria.

**Table 2:** Criteria for considering studies for the review based on the Population, Intervention, Comparator, Outcomes, and Study Designs (PICOS) structure.

	Population	Intervention	Comparator	Outcomes	Study designs
Inclusion criteria	Aged 5–17 years; no LUT symptoms or bowel elimination problems; no cardiovascular and neurological diseases; no hypercalciuria; no organic urological disease and so on	Desmopressin or desmopressin + placebo	Desmopressin + anticholinergic agent	FR; FR + PR; the mean number of wet nights and side effect	RCT
Exclusion criteria	Children with known contraindications to NE treatment, as assessed by a doctor; patients aged <5 years and >18 years; a combination therapy of desmopressin plus other drugs (not anticholinergic agents); and others	Other therapies	Other therapies	Qualitative outcomes such as inadequate indicators and others	Observational study, letters, comments, reviews, and animal experiment

## Efficacy

### One month

#### FR

**Figure 4:**
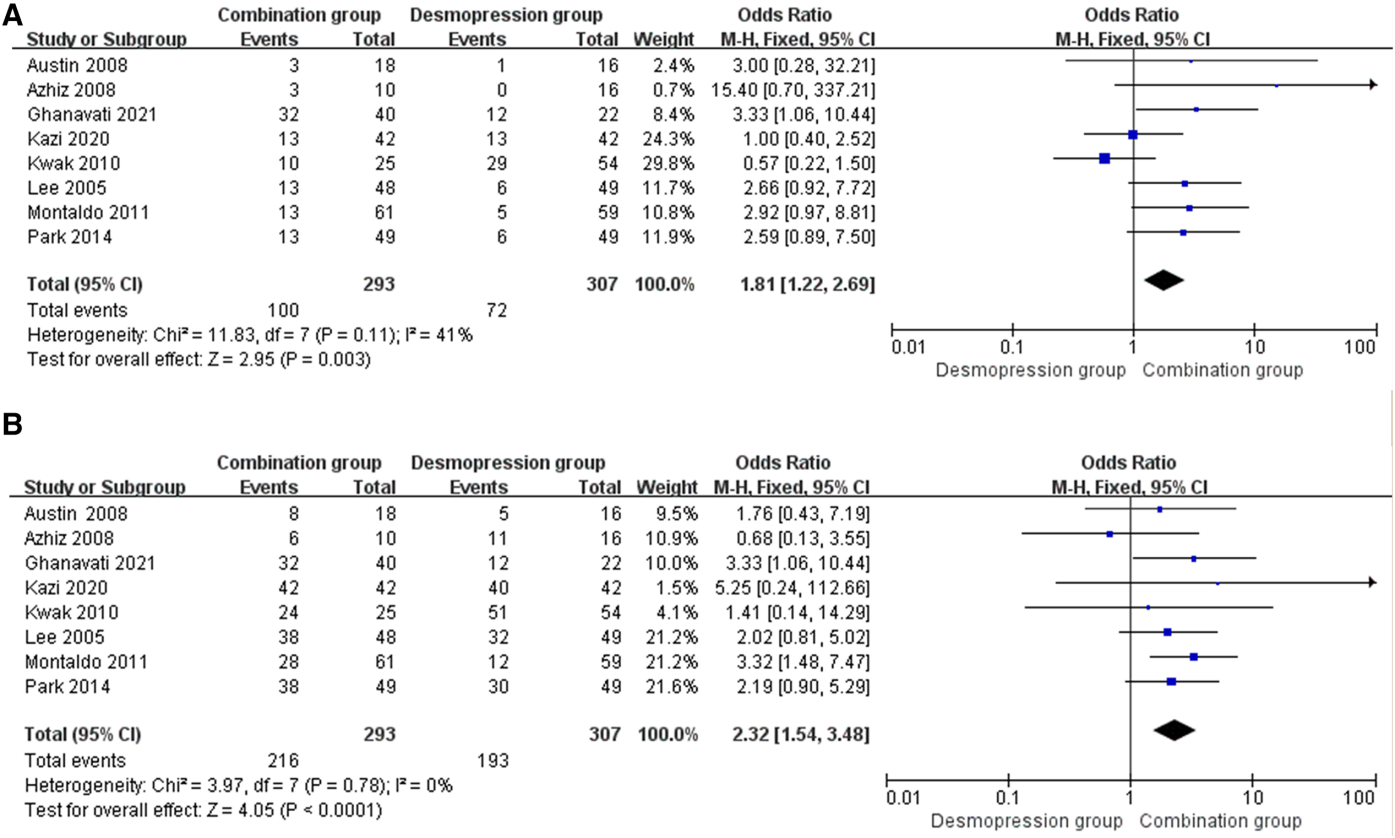
Forest plots showing changes between two groups in (**A**) FR at 1 month and (**B**) FR+PR at 1 month.

Eight studies involving 600 patients (293 in the combination group and 307 in the desmopressin group) contained meaningful data. A fixed-effects model was used to evaluate changes between the two groups, which showed an OR of 1.81, 95% CI: −1.22 to 2.69, *P* = 0.003. The result of the research proves that the combination group showed more significant improvement in FR compared with the desmopressin group (Figure 4A).

#### FR+PR

Eight studies involving 600 patients (293 in the combination group and 307 in the desmopressin group) contained meaningful data. A fixed-effects model was used to evaluate changes between the two groups, which showed an OR of 2.32, 95% CI: 1.54–3.48, *P* < 0.0001. The research results proved that the combination group showed greater improvement in FR+PR compared with the desmopressin group (Figure 4B).

### Three months

#### FR

**Figure 5:**
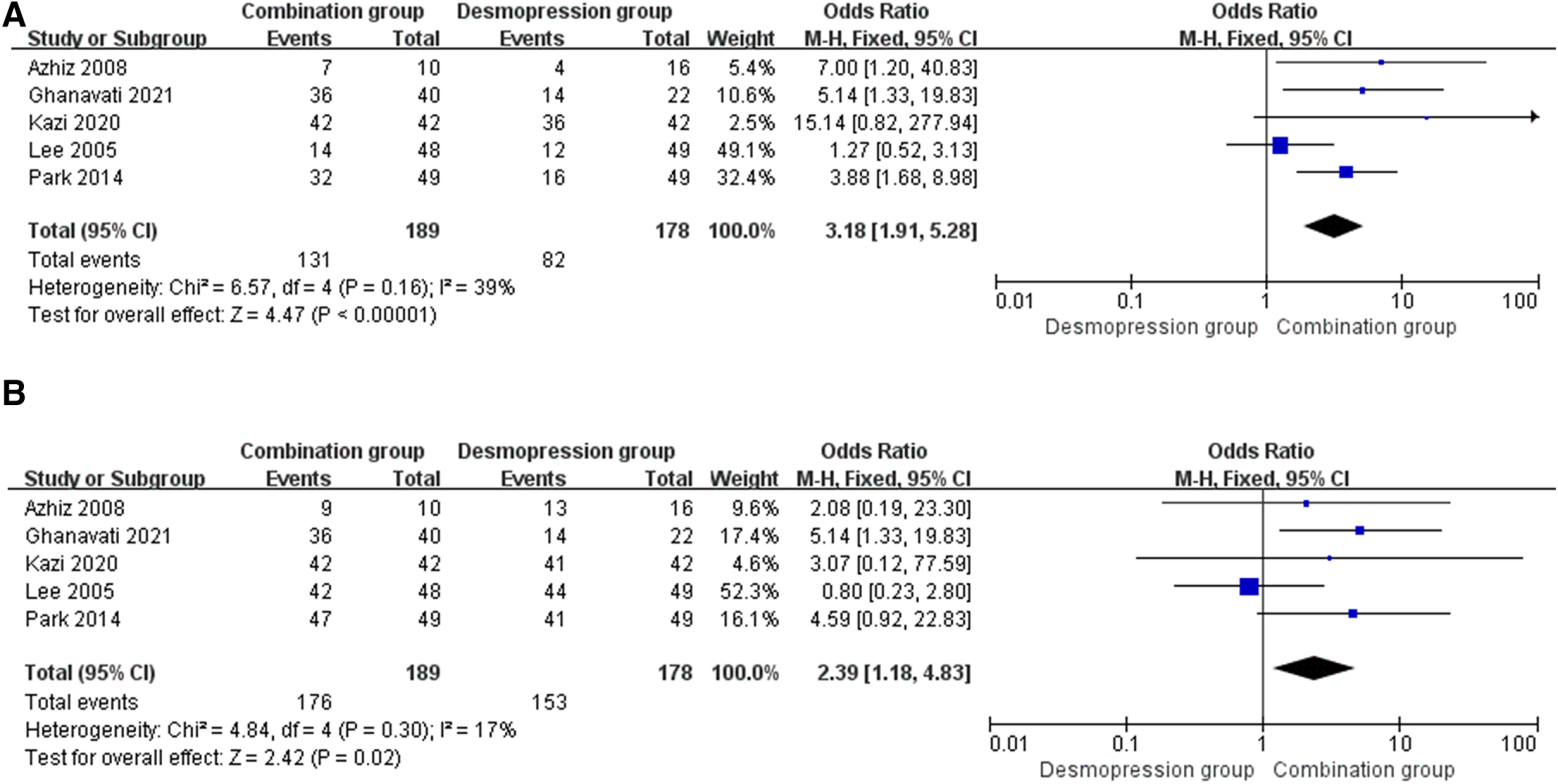
Forest plots showing changes between two groups in (**A**) FR at 3 months and (**B**) FR+PR at 3 months.

Five studies involving 367 patients (189 in the combination group and 178 in the desmopressin group) contained meaningful data. A fixed-effects model was used to evaluate changes between the two groups, which showed an OR of 3.18, 95% CI: 1.91–5.28, *P* < 0.00001. The result of the research proved that the combination group showed greater improvement in FR compared with the desmopressin group (Figure 5A).

#### FR+PR

Eight studies involving 367 patients (189 in the combination group and 178 in the desmopressin group) contained meaningful data. A fixed-effects model was used to evaluate changes between the two groups, which showed an OR of 2.39, 95% CI: 1.18–4.83, *P* = 0.02. The research results prove that the combination group showed greater improvement in FR+PR compared with the desmopressin group (Figure 5B).

## The mean number of wet nights

### One month

**Figure 6:**
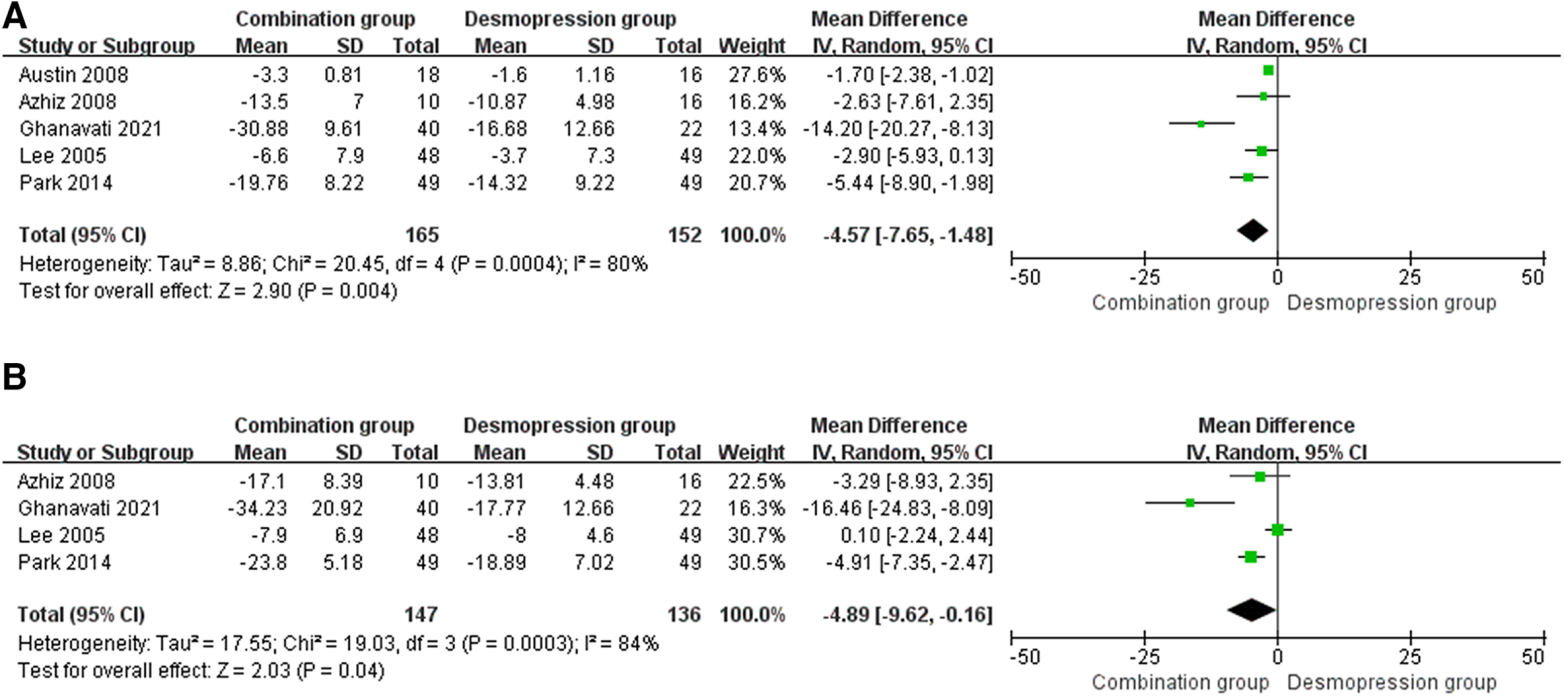
Forest plots showing changes between two groups in (**A**) the mean number of wet nights at 1 month and (**B**) the mean number of wet nights at 3 months. IV, inverse variance.

Five RCTs involving 317 patients (165 in the combination group and 152 in the desmopressin group) recorded changes in impact in the mean number of wet nights (Figure 6A). A fixed-effects model showed an MD of −4.57, 95% CI: −7.65 to −1.48, *P* = 0.004. The results suggest that the combination group showed statistical differences in the impact on the mean number of wet nights compared with the desmopressin group.

### Three months

Four RCTs involving 283 patients (147 in the combination group and 136 in the desmopressin group) recorded the changes in impact in the mean number of wet nights (Figure 6B). A random-effects model showed an MD of −4.89, 95% CI: −9.62 to 0.16, *P* = 0.04. In addition, there were differences in the mean number of wet nights between the two groups.

## Safety

### Side effect

**Figure 7:**
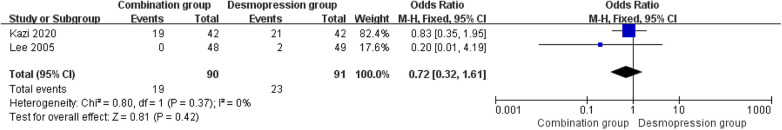
Forest plots showing changes between two groups in side effect.

Two RCTs, including 181 participants (90 in the combination group and 91 in the desmopressin group), were involved in the research for side effect. The study of the OR was 0.72, and the 95% CI was 0.32–1.61 (*P* = 0.42). These results indicate that there is no significant difference between the two groups in terms of side effect (Figure 7).

## Discussion

NE, also known as enuresis, is a common problem for children. The prevalence of NE is falling by approximately 15% a year, and the incidence rate for boys is higher than that for girls (26). A great lot of evidence sustains that NE is a heterogeneous disease. Several important etiological factors can explain this disease. The genetic determinant is one of the important mechanisms that cause NE. Children had a 44% and 77% increased risk of having the disease if one or both of their parents suffered from NE (27). Nevertheless, no specific gene that appeared to be involved in NE had been discovered until now (27). Disturbed and delayed maturation plays an important part in the pathogenesis of NE (28). Although desmopressin is the first choice for treating NE, the treatment alone is not satisfactory sometimes. Anticholinergic therapy has been proven to be an effective solution for children who no longer respond or have partial responses to desmopressin monotherapy.

The systematic review and quantitative meta-analysis showed evidence from RCTs regarding the efficacy and safety of desmopressin compared with anticholinergic agents for NE. For the study, the inclusion criteria included patients aged between 5 and 17 years, no neurological or urological cause for enuresis, and no LUTS or bowel elimination problems. In a 1-month-long study, compared with the desmopressin group, the combination group showed statistical difference in FR (*P* = 0.003) and FR+PR (*P* < 0.0001) for NE. During the 6-month study, the combination group also showed statistical difference in FR (*P* < 0.00001) and FR+PR (*P* = 0.02) compared with the desmopressin group for NE. Meanwhile, for the change in the mean number of wet nights, the combination group showed statistical differences compared with the desmopressin group for NE at 1 (*P* = 0.004) or 3 (*P* = 0.04) months. To sum up, the effect of combined medication is more effective, which is superior to the alone group.

Desmopressin monotherapy is often considered the first-line treatment for NE. It can reduce urine production in the nighttime (29). Desmopressin is relatively safe and effective; reports have claimed that the effective rate is between 60% and 70%. Approximately 30% of patients are FR, and 40% partially responded to the treatment (30, 31). Meanwhile, patients with oral desmopressin should limit water intake in the evening because of its side effects (water intoxication and hyponatremia). As mentioned earlier, using a single drug has failed to achieve satisfactory treatment outcomes. Therefore, anticholinergics combined with desmopressin in treating NE was regarded as second-line therapy for patients who failed to respond to desmopressin monotherapy (23, 32). It has been confirmed that anticholinergic treatment can reduce the frequency and severity of urge incontinence in children with non-organic lower urinary tract (LUT) dysfunction (33). Meanwhile, studies have pointed out that anticholinergic agents may play an essential role in improving the vesical volume and thickness of the bladder wall for patients with NE (24). In our study, the alleviation of symptoms is much better in the combination therapy than the desmopressin monotherapy. Thus, the success of combination therapy ultimately depends on two pivotal regulatory factors: (1) desmopressin can decrease the production or secretion of nocturnal urine (34, 35) and (2) anticholinergic agents can increase bladder capacity and reduce detrusor overactivity (36). It is noteworthy that the anticholinergics monotherapy is not currently recommended (37).

Only two studies reported on the results of adverse events (AEs). Significantly, the incidence of adverse reactions between the two groups (*P* < 0.42), such as headache, nausea, and decreased appetite, was not significantly different. Therefore, these results demonstrate the safety of desmopressin in conjunction with anticholinergics in treating NE. Also, note that the study excluded children with constipation; the anticholinergic treatment may worsen the symptoms of constipation, and the rate of this side effect is 0.2%–2.3% (38). In addition, the potential mechanisms of combination therapy are currently unclear and need further studies.

In a word, this meta-analysis included eight RCTs and concentrated on the efficacy and safety of desmopressin in combination with anticholinergic agents for treating NE. This study had advantages compared with previous studies. The data of this meta-analysis were derived from randomized, double-blind, controlled trials. Thus, the results of this analysis can provide a basis for guiding clinical applications. However, this study also has some limitations. The doses of desmopressin and the types of anticholinergic agents were not completely the same in this article, which may affect our meta-analysis quality. Therefore, our research will need more appropriate high-quality randomized trials to improve the accuracy of results.

## Conclusions

In summary, this meta-analysis elucidates that desmopressin combined with anticholinergic agents is more effective in treating NE than desmopressin monotherapy. Both methods are noted to be safe, and the anticholinergic agent does not increase the risk of AEs.

## Data Availability

The original contributions presented in the study are included in the article, further inquiries can be directed to the corresponding authors.
